# Clinical epidemiology: A daydream?

**DOI:** 10.1007/s10654-017-0226-2

**Published:** 2017-01-24

**Authors:** Jan P. Vandenbroucke

**Affiliations:** 1 Department of Clinical Epidemiology, Leiden University Medical Center, PO Box 9600, 2300 RC Leiden, The Netherlands; 2 Department of Clinical Epidemiology, Aarhus University Hospital, Aarhus, Denmark; 3 London School of Hygiene and Tropical Medicine, London, UK

The leitmotif of my inaugural address at Leiden University in 1986 [1] was a quote by the first professor of epidemiology at the London School of Hygiene and Tropical Medicine, Major Greenwood. In 1936 Greenwood described how 100 years earlier, in the first half of the nineteenth century, there had been a movement in France, the so-called ‘Médecine d’Observation’, i.e., ‘Observational Medicine’. The figurehead of that movement, Pierre Charles Alexandre Louis, argued that one needed to study large groups of patients and draw numerical conclusions as a basis for the theory and the practice of medicine. Louis’s fame lives on because of a report where he tried to show that bloodletting was of no avail in patients with pneumonia [2]. Greenwood, however, described how the movement had never made it—it had disappeared from the clinic, and he concluded with a lament: *“If only Louis had succeeded in really commanding the support of… the great clinical teachers of Paris, if Trousseau had had a service statistique and Dieulafoy!… I dare say that by now* [in 1936] *the Royal Colleges would be considering the desirability of establishing a Diploma in Clinical Statistics and clinical units would have statisticians. But this is mere day*-*dreaming.*” [3].

That was my daydream, too—it was my motivation to accept the Chair of Clinical Epidemiology at Leiden University in the Netherlands in 1986.

Over the past 30 years, numerical research on groups of patients has taken off spectacularly [4]. By ‘numerical research’ I mean research in which persons (e.g., patients) are the unit of observation and characteristics, or outcomes, are counted in groups of persons. The upsurge of numerical research happened really quickly. ‘Evidence-based Medicine’, the movement that is in favour of basing medical practice on numerical data, and the ‘Cochrane Collaboration’, which advocates that these numerical data should be combined in systematic literature reviews, have become household words for everyone with a medical education. Yet, the notions ‘Evidence-based Medicine’ and ‘Cochrane Collaboration’ as we know them today did not exist at the time of my inaugural address. Only in 1992 these names were introduced in medical journals and it was described what they stood for [5, 6].

In the first part of this paper, I will describe the way in which micro-history, i.e., my own career and the department clinical epidemiology at Leiden, The Netherlands, are interwoven with this macro-history. This aims at giving a glimpse of how clinical epidemiology was introduced and became influential in one European country. It is of necessity steeped in local and personal history.

The next two parts will treat problems that preoccupied me over the years. Firstly, the problem of the oversimplification of the clinical application of epidemiology, *the problem of ‘cookbook epidemiology’.* Secondly, my concern about a possible dismantling of science; *the battle for the soul of science.*

In a brief third part, I will look at what work is still left to do to make Greenwood’s daydream come completely true.

## Micro-history and Macro-history

My first encounter with epidemiology was in an optional course during my medical training in Leuven, Belgium, in 1971. I was immediately and completely captivated. I liked the ‘helicopter view’, the idea that you can reflect on diseases in entire populations in size and number; that you are able to understand how and why the number of cases of a disease is high or low in a population, and how this may change in the course of time. This was a line of thinking that was entirely different from all the other courses in the medical curriculum.

After my medical training, I specialized in internal medicine. Halfway through the specialisation, I became restless and longed for more insights. By chance, I saw an announcement for The Ten Day International Teaching Seminar in Cardiovascular Epidemiology, in Denmark in 1976—organized by Rose and Jeremiah Stamler, with Geoffrey Rose, Dick Remington and Henry Blackburn on the faculty. When the course was over, I had made my decision: becoming an epidemiologist was the greatest thing in the world.

I took more epidemiology courses, including a new one that was organised by the Dutch Heart Foundation in 1977, taught by a leading academic from the Harvard School of Public Health, Olli Miettinen. He presented new viewpoints on old epidemiological notions and suggested I come to Boston for further training. At Harvard, in 1978–1979, I got to know the content of epidemiology. This was one great intellectual feast. But, I also came across ideas *about* epidemiology. There was talk about a new movement, which was only a few years old, to bring epidemiology back to the clinic. The story that went with it was that a rift had developed between ‘Schools of Public Health’ and ‘Schools of Medicine’. Schools of Public Health had been founded in the nineteenth century and the beginning of the 20 st century. All the numerical thinking about health and disease and about causes and evolution of frequencies of disease had ended up in the Schools of Public Health. It had disappeared from Schools of Medicine. Only basic scientific research and pathophysiological research were practised in Medical Schools and offered as the scientific basis of medicine. This had to change—it was said—because medicine had become increasingly powerful, expensive and complex. There was a need for more knowledge in size and number about patients, their histories and the clinical course of their diseases, as a guideline for medical reasoning about aetiology, diagnosis, prognosis and interventions. Thus, it was held necessary to found departments of clinical epidemiology. The Rockefeller Foundation and the Robert Wood Johnson Foundation supported these ideas. The atmosphere was rife with slogans such as ‘Clinical Epidemiology, a basic science for clinical medicine’ and ‘Clinical Epidemiology, the architecture of clinical medical research’—which became titles of highly influential textbooks [7, 8].

These ideas immediately tied in with my thinking and my preoccupations: this was what I wanted to devote myself to.

After my training in Boston, I enthusiastically approached medical schools in my home country, Belgium, and proposed to found a combined centre for statistics, epidemiology and informatics (still in its infancy in medical applications at the time)—with the express purpose of assisting clinicians in conducting clinical research. There was no interest. Miettinen suggested to enquire in the Netherlands, with the Netherlands Heart Foundation. In the Netherlands, medical charity funds were among the first institutions to actively start supporting epidemiology in its clinical applications. The then medical director of the Netherlands Heart Foundation, had established contacts at the Harvard School of Public Health, which was the reason why Miettinen had started to give annual courses in the Netherlands, –of which I had followed the first. Due to his courses and influence, various epidemiologists and health professionals from the Netherlands were trained at the Harvard School of Public Health already in the 1970s, and even more in the 1980s and 1990s.

The Netherlands Heart Foundation offered me a job to help grant applicants to write up better research proposals. One was Hans Valkenburg, head of epidemiology at the Erasmus University Rotterdam—originally an internist and microbiologist, trained in population epidemiologic research in the United States. Valkenburg had been brought to Rotterdam by the founding dean of the Rotterdam medical school, Andries Querido. Querido became interested in ‘community medicine’ in the 1960s: the idea was that one had to understand diseases in the community in which they originate. Upon the foundation of the new Rotterdam medical school, he made sure that an independent department of epidemiology was included [9]. Valkenburg provided me with a great deal of critical feedback about the teaching I had received at Harvard—and I thought him all the more sympathetic for it. After just over a year, in 1981, I switched to his department.

Ideas from the other side of the ocean had an increasingly strong impact in the Netherlands. At the Amsterdam Medical Center, there was a strong influence from the Clinical Epidemiology department at McMaster, Canada, amongst others via Harry Buller who had trained there when he was still a resident. We charged around the Netherlands with a group of enthusiasts, comprising also Bert Hofman and Koos Lubsen from Rotterdam. We organised evenings at university hospitals, with exercises on reading clinical research—without any funding and without any association with any training scheme—just in everybody’s spare time—because it was so much fun and so new. In 1984, a brainstorming session was organised about the future of clinical epidemiology in the Netherlands, attended by a representative of the Rockefeller Foundation [10].

Suddenly, there was interest everywhere. The scarce epidemiologists were offered chairs in several universities in the Netherlands. Under the supervision of Hans Valkenburg from Rotterdam, with help of the head rheumatology at Leiden University, I had gained a doctorate in 1983 with research about a possible protection from rheumatoid arthritis by the use of oral contraceptives [11, 12]. This paved the way. Leiden University Medical School decided to dismantle the department of social medicine, which was thought to be ‘old’ and replace it with the ‘new’ clinical epidemiology. Three years after my doctorate, in September 1986, at the age of 36, I found myself in the brand new building of the current Leiden University Medical Center, as the head of an empty department—all on my own in three rooms, next to the hospital beds storage, sitting on cardboard boxes. This unusual place was the result of the fact that I had insisted on being located within the university hospital, and not somewhere in a far-away ‘research’ building. It had to be easy for clinicians to walk in.

Immediately I established contacts with clinical departments in order to find topics that enabled me to demonstrate what the clinical application of epidemiology was good for. Three months after my arrival in Leiden, I met a clinician doing research on venous thrombosis. He told me they had reached a dead end in the research into rare biochemical abnormalities that might be the cause of deep vein thrombosis. Patients who developed thrombosis at an early age, especially when this happened several times in one family, were sent to Leiden from all over the Netherlands for elucidation of possible biochemical abnormalities. Yet, internationally, it was strongly doubted whether all of the, mostly rare, hereditary abnormalities which were found were a good explanation of the causes of the thrombosis in these patients. My immediate response, based on my methodological training, was to tell my Leiden colleagues to stop collecting ‘rare postage stamps’. Rather than conducting research with patients referred from far and wide, I proposed to set up a case–control study with successive patients as they presented in daily practice, with an accompanying control group from the population—and to determine the presence of their favourite coagulation abnormalities in both groups. In itself, this design was a major innovation within coagulation research. The research was set up in intensive cooperation with clinicians and basic scientists. The rest is history: the discovery of factor V Leiden [13], the interaction of factor V Leiden with ‘the pill’ [14], the ‘oral contraceptive controversy’ with an increased risk of vein thrombosis due to newer contraceptives [15], the research into the effect of coagulation mutations in the past, until well in the nineteenth century [16, 17].

In addition to coagulation, the Leiden department of clinical epidemiology occupied itself with a great many other subjects: psychiatry, nephrology, general internal medicine, endocrinology, skin diseases, geriatric medicine and rheumatology. There were breakthroughs in several areas—always in collaboration with physician-researchers, often together with basic scientist. By way of example, we did research into the presence of antibodies in patients with rheumatoid arthritis, who had been blood donors long before becoming patients, and in whom the antibodies were detected in their stored blood—long before they developed the disease [18]. Diversity is often the fate of the epidemiologist. My hero Greenwood once wrote that he himself had not discovered that much new, but had helped others to discover new things [19]. I also often look back with a great deal of joy on things that I influenced indirectly, such as the research into the inverse relationship between longevity and fertility—based on historical data from the British nobility [20].

One aspect became gradually clear: the clinical application of epidemiology as it developed in Leiden differed from that of other types of clinical epidemiology. When Evidence-Based Medicine and the Cochrane Collaboration movements originated at the beginning of the 1990s, the application was mainly in research into diagnosis, prognosis and therapy; not in aetiology, the causes of disease—nor in pathogenesis, how these causes interact with the organism. The focus of Leiden epidemiology was precisely these—in particular about disease processes that were of interest to third line physicians working at university hospitals. Following a working visit, Alvan Feinstein told me that we practised ‘pathophysiological epidemiology’. In 1989, I described some guiding principles of the Leiden department [21].

We founded a ‘school’, and that was precisely the intention. With my close colleague and later successor as the head of the department, Frits Rosendaal, we established a clinical epidemiology course on a remote spot in the Netherlands (a small island a few kilometres offshore). With the department of internal medicine we set up an epidemiology internship for residents in internal medicine. It was one of the most coveted internships. Many of the residents who followed this internship ended up as training supervisors for residents, department heads and chairpersons of national committees. This is not an experimental observation, of course: the residents who chose this internship probably already had wider interests, but it is a joyous observation all the same.

In this first part, I have tried to make clear how my career was propelled, mixed with and sometimes challenged in an international ‘clinical epidemiology’ movement. This description does not answer the question *why* numerical thinking broke through in the clinic, and why this did not happen before, in particular at the beginning of the nineteenth century around the time of Médecine d’Observation [4, 22, 23]. This needs further reflection from medical historians [24].

In these developments, I came across a number of problems about which I entered into extensive debates: the battle against *cookbook epidemiology* and the battle for the *soul of science.*

## Cookbook epidemiology

Evidence-based Medicine originated from clinical epidemiology in the 1980s and early 1990s. It was to be a radical break with the past: no longer was medicine to be based on mere expert opinion and on half-baked ideas from basic sciences, but it should be based on an ‘objective’ numerical foundation by directly counting what happens to people. The most objective research, it was thought, was the randomised controlled double blind trial. This led to the idea of a hierarchy of evidence, with the randomised trial right at the top, and all non-experimental forms of research, such as observational research in its various forms further down—as these were considered to be increasingly suspect.

This hierarchy was a thorn in my side from the word go. Medical practice is not based only on results of experiments on diagnostics or therapy. There are a great many important insights that you cannot test with a randomised experiment: think of the transmission of infectious diseases, e.g., think of HIV; needless to say, no experiments have been carried out on its transmission in people—and yet we believe observations from which we deduce how this virus is transmitted—global campaigns are based on this knowledge. Think also about genetic knowledge that increasingly leads clinical decisions and is inherently observational.

My argument was and is that you need all forms of research—*which* type of research depends on the research question. Depending on the research question, all types of research are of equal value [25, 26].

However, this debate about the hierarchy of evidence is now the debate of the twentieth century. It has been replaced with a new debate in the twenty-first century, about a new form of simplification that goes much further. This new movement calls itself ‘causal inference’ [27]. One basic idea of this movement is that you can only make causal statements about interventions. Causing something presupposes that you do something, perform an intervention. This is always the case in a randomised trial; after the randomisation, you set up an intervention in one group, and a different one in the other group. The new movement argues that observational research may be just as worthwhile as randomised trials—which seems an improvement compared to Evidence-Based Medicine. Yet, it argues that observational research can only demonstrate and estimate causality when interventions are involved. What is wrong with this? One consequence is that what is not an intervention, but a ‘state’, can no longer be researched as a cause [28, 29]. For example, you can no longer compare the survival rate of a group of fatter people to that of a group of thinner people, and decide that being fat *causes* a shortened life expectancy, because being fat or thin are not interventions. The same would then apply to the state of high blood pressure, or high cholesterol, or having diabetes: you cannot say anything causal about these ‘states’. They are not interventions and fall outside this so-called ‘interventionist view’ of causes. However, there are more views regarding causality: next to ‘interventionist causality’ there are ‘etiological or historical causes’ as well [30]. These are causes that provide an insight into how something comes about, and this may involve ‘states’—on which we might currently not be able to intervene—but where we might be able to intervene at some point in the causal chain in the future, and which are important to understand how diseases come about. The understanding of what a cause is, is far too limited in this new thinking. It is not very smart to rely on one type of causality for epidemiology. We need various forms of causality.

Why am I fussing so much about these very abstract reasonings on causality? Because in this new thinking, it suddenly looks as though a great deal of research about how diseases originate is no longer legitimate and is somehow second rate. This disregards the actual power of epidemiology.

The essential problem of ‘cookbook thinking’ is that solutions are only sought within rules and procedures of one type of knowledge: the methodology of numerical research. When you have ticked off all the methodologic rules, you may decide that something is a cause. This situation is very similar to that of the story of Baron von Munchausen, who wanted to pull himself out of a swamp by pulling on his own hair. This does not work: in order to get out of a swamp, you need a lever, a lever from outside. That is exactly the same in science. In *all* sciences. The basic scientist who looks for an explanation for the carcinogenic effect of tobacco smoke, does so because of background knowledge from another science: epidemiology. If she did not have epidemiological knowledge about smoking and lung cancer, it would be foolish to search for a carcinogenic mechanism related to tobacco smoke [31]. Thanks to epidemiology, she can even afford to throw all negative findings away, until she discovers something that seems carcinogenic, for there must be a mechanism. Epidemiology as a science deals with occurrence research in groups of people. Epidemiology also needs levers: data can only be collected and interpreted in the light of a story about how things come about. Deciding that something is a cause requires a judgement in which one brings together results from various types of research, and various types of science. This becomes a weighting of methodologic arguments, of the exclusion of alternative explanations, of background knowledge from basic-science and pathophysiological mechanisms. There are no rules for this weighting. A judgement that something is a cause always remains a risk; it is a type of ‘best guess’, based on currently available information. This feels uncomfortable. People try to escape this situation with box-checking strategies. But it remains inescapable: judgement originates through integration and weighting of relevant research and theory.

Some of the original objections that I and others have made to this type of ‘causal inference’ [32], and answers by the originators of this movement [33, 34] have led to an array of papers, published in the December 2016 issue of the International Journal of Epidemiology. Interested readers can read all arguments and counterarguments in full in several papers in that issue.

## The battle for the soul of science

Science is fragile. Science is fragile because it is based on an agreement between people about how to organise the acquisition of knowledge.

The nineteenth century American philosopher Charles Peirce described how there are various sources of knowledge: tradition, authority, dogma and science. He argued that science is the best source because it means that you jointly search for the right answer, in mutual debate, based on investigations (i.e., research). We agree these days that science only exists when research is published publicly and discussed publicly. No research is perfect, and it is possible to criticise any research, but this does not matter—this is the very thing you have to argue about. It is precisely because of the imperfection of the previous research that you set up new research. Consensus originates from the discussion; it is never final, is subject to continuous change and is never complete. This view of science allows to put our day-to-day activities into perspective. Some scientific investigations may be on the wrong track for a long time. Yet we argue that science is ‘self-cleansing’ in the long run—admittedly, sometimes in the *very* long run. ‘Self-cleansing’ is *not* to say that we will eventually know how reality works, but it means that ideas that are in conflict with reality will perish in the shorter or longer run. But, that can only happen if the principle of a public debate based on public investigations is followed.

A great deal can go wrong. Many problems arise under the influence of external factors, where *profit* plays an important role. This is most clearly visible in research set up by the pharmaceutical industry. Research by one manufacturer nearly always ends up in favour of their own product and to the detriment of the competitor [35, 36]. Please note that all research—and this includes basic scientific research—has a tendency to move towards results that are believed by the researcher. The reason is that research is ‘theory-loaded’: in setting up a study, the concerns and judgements of the researcher play an important role. That leads to a great many minor and major decisions about the type of patients, the type of outcomes, the way in which you set up the comparison, the duration of the research, etc.; all these decisions influence the outcome and interpretation of research [35, 36].

The solution is not just public debate in itself. Public debate is best served by new research that is set up differently by critics of the old research. Unfortunately, that is no longer possible for research into products of the pharmaceutical industry, because there is no funding for such separate research—the industry itself exclusively decides what research is carried out with its products, and how it is carried out. It is not unthinkable that successive new products push out older ones, and are slightly less good in some respects—but that we will never find out. To find out, we need research that is set up differently by different persons. Please note that this is not to say that the research of the critic always survives and that the research of the industry is always wrong. But in order to know which research survives, both research undertakings—funded by industry and not funded by industry—have to be present. As research into new medicines is set up and carried out almost exclusively by the pharmaceutical industry, a full and worthy debate about the merits of new drugs is no longer possible. Then science ceases to exist [37–39].

The problems are not just about money. Another threat is excessive ‘autonomy’ thinking. Opposition to it brought me in debates with health lawyers, ethicists and persons concerned with privacy. My starting point was that for centuries, physicians have been learning from what they experience with patients; they learn lessons from what occurs to their patients in practice. This is one of the sources of progress in medicine. To me, it logically follows that this is a sufficient reason, and a compelling argument, to use the stored data and material of patients who have already been treated in the past in order to learn from the experience with these previous patients for patients in the future. This idea is increasingly at odds with the norm of absolute autonomy where patients need to be asked if this is permitted [40, 41]. I argued against this tendency on the basis of the principle of solidarity, and also because of the idea that no personal damage whatsoever is caused to patients by the use of past data; on the contrary, patients are often enthusiastic that their data, their suffering, will be used for new insights that may help others. It has repeatedly been demonstrated that research with existing data where individual permission is asked, leads to false results because it is precisely the people who are faced with an unpleasant outcome who respond negatively when you ask permissions (see multiple references in Ref. 36). In the past decade, this battle has moved to the European level.

Open science, as described by Peirce is also under other pressures: first, by the exceedingly competitive selection mechanisms that are forced on young scientists during grant application processes, because of a dearth of financing—and second, because at the same time more and more often the results of science are not accepted and denied for political, ideological or economic reasons. The central question remains how self-evident it is that the ideal of an ‘open science’ will continue to exist. It is an agreement amongst humans about the organisation of the acquisition of knowledge. This agreement originated in a certain era, many centuries ago. It is a cultural expression that requires an effort to keep it up, in particular when the results are not always easily accepted. Cultural expressions may disappear. It takes vigilance to ensure that they continue to exist [42].

## The future of Greenwood’s daydream

In his 1936 daydream, Greenwood stated that the clinical application of statistics would be institutionalized as a diploma, awarded by the Royal Colleges whose task it is to register medical specialists in the UK. What is the current situation of the combination of clinical medicine with epidemiology?

The picture is mixed. Some years ago, the departmental head of one department of epidemiology of a School of Public Health in the US told me: “We are the last of a dying species”. By ‘dying species’ he meant: the physician-epidemiologist. In his department, there were no longer physicians who follow the epidemiology masters course, let alone the PhD. In his view, clinical departments need to employ PhD epidemiologists for the supervision of clinical research. In contrast at other departments of epidemiology and other schools of public health in the US, separate course in epidemiology exist for clinicians, some already with a long tradition. Also in European countries the situations is mixed. In the Netherlands there is a large influx of non-medical Master and PhD students in the formal epidemiology training programmes. In Denmark, I still see many medical students and young medical doctors doing epidemiologic research before or during clinical residency training programs.

My opinion remains that we need people with both types of knowledge in one head. The same brain should understand both worlds: the clinic and methodology. This leads to creativity in setting up research and in the development of new methodologies that are required to solve new medical problems. People with both types of knowledge are able to talk to pure clinicians as well as to pure methodologists, and both conversations will run more smoothly and have a better quality—which will lead to better research. Clinicians with methodological knowledge are almost the only ones still capable of in-depth reading of clinical research. To the pure clinician, the clinical research published in general medical journals is no longer really understandable, because the design and the analyses have become too complicated. To the pure methodologist, it may not be evident how particular findings fit with background clinical and basic science knowledge. People with both types of knowledge are the ones that can make or break clinical guidelines because they are able to adopt a critical attitude towards the research behind the guidelines, and at the same time they understand the way of thinking of clinicians and the intricate organization of health care. If we want to preserve and reinforce proper judgements about the theory and practice of medicine—including the use of ‘big data’ and the use of ‘personalised medicine’—we need people with both types of knowledge in one head.

Meanwhile, the large, enthusiastic tidal wave of clinical epidemiology, on which I have surfed for the past 30 years, together with many others, has stopped raging, and it is now quietly lapping on the beach. The future will show what solutions will be found for training, for career opportunities, and for the positioning of clinicians with methodological knowledge in the wider progress of medicine.

## Electronic supplementary material

Below is the link to the electronic supplementary material.

Supplementary material 1 (PDF 727 kb)
