# Supplementary material for: Clinical epidemiology: A daydream?
Source: Eur J Epidemiol. 2017 Jan 24;32(2):95–101. doi: 10.1007/s10654-017-0226-2 (PMC5374175; doi:10.1007/s10654-017-0226-2)
Supplement: Supplementary file 1 — Supplementary material 1 (PDF 727 kb) [file 10654_2017_226_MOESM1_ESM.pdf]

Clinical Epidemiology: a daydream? *

Valedictory Lecture, Jan P Vandenbroucke

5 June 2015

Leiden University, The Netherlands

*Translated by translation bureau "Uit de Kunst" http://uitdekunst-vertalingen.nl/

Amended by Jan P Vandenbroucke during August 2015

"If only Louis had succeeded in really commanding the support of… the great clinical teachers of Paris, if Trousseau had had a *service statistique* and Dieulafoy!... I dare say that by now the Royal Colleges would be considering the desirability of establishing a Diploma in Clinical Statistics and clinical units would have statisticians. But this is mere day-dreaming."

Major Greenwood. "Louis and the numerical method'. IN: The medical dictator and other biographical studies. London, Williams and Norgate, 1936, pages 123-142. Reprinted with an introduction by Austin Bradford Hill, The Keynes Press, British Medical Association, 1986.

The Rector, members of the Board, dear colleagues, ladies and gentlemen,

28 years ago, I held my inaugural address in this hall (1). The leitmotif of my address was a quote by the first professor of epidemiology at the London School of Hygiene and Tropical Medicine, Major Greenwood.[i]

In 1936, Greenwood described an event that took place 100 years before. In the first half of the 19th century, there was a movement in France, the so-called 'Médecine d'Observation', i.e., 'Observational Medicine'. The figurehead of this movement was Pierre Charles Alexandre Louis. Louis argued that one needed to study large groups of patients and draw numerical conclusions to support the theory and the practice of medicine.

Greenwood described how this movement had never made it - it had disappeared, and he concluded with a lament: *"If only Louis had succeeded in really commanding the support of… the great clinical teachers of Paris,... I dare say that by now* [in 1936] *the Royal Colleges would be considering the desirability of establishing a Diploma in Clinical Statistics and clinical units would have statisticians… but, this is mere day-dreaming*."(2)

This was my daydream, too – it was the motivation with which I started in Leiden in 1986, upon accepting the Chair of Clinical Epidemiology.

Over the past 30 years, numerical research on groups of patients has taken off spectacularly. The journals are full of it. It happened really quickly. All of you know 'Evidence-based Medicine', the movement that is in favour of medical practice being based on numerical data. You also know the 'Cochrane Collaboration',[ii] which advocates that numerical data be combined in systematic literature reviews. The notions 'Evidence-based Medicine' and 'Cochrane' did not yet exist at the time of my inaugural address. It was not until 1992 that they were introduced for the first time in medical journals (3, 4).

In the first part of this lecture, I will describe the way in which micro-history, my own career, and Leiden Clinical Epidemiology are interwoven with this macro-history.

In the second part, I will speak about two problems that preoccupied me in these developments. Firstly, the problem of the over-simplification of the clinical application of epidemiology, *the problem of 'cookbook epidemiology'.* Secondly, my concern about a possible dismantling of science; *the battle for the soul of science.*

In a brief third part, I will look at what is still left to do to make *Greenwood's daydream* come completely true.

Micro-history: how did I encounter epidemiology?

During an optional course in the fourth year of my training to become a physician in Leuven, Belgium – 1971. The optional course was 'Epidemiology', taught by the professor of cardiology.[iii] Why did I choose this optional course?

It was the only optional course for which you did not need to take an examination. It was enough to simply attend. Yet after several minutes, I was sitting on the edge of the wooden bench and starting to take notes. I was entirely captivated. I jotted down everything, and I still have my notes.

What inspired me in this course? The helicopter view, the idea that you can reflect on disease in entire populations in size and number; that you are able to understand how and why the number of cases of disease is high or low in a population, and how this may change over the course of time. This was a line of thinking that was entirely different from all the other courses in the medical training.

After the medical training courses, I began the specialisation in internal medicine. Halfway through the specialisation, I became restless. I started asking myself questions about whether it would not be much better to prevent diseases in the population. And I was of the opinion that we knew far too little about the course and outcome of diseases.

By chance, I saw an announcement for an international course in the 'epidemiology of cardiovascular diseases' in Denmark in 1976.[iv] The professor with whom I had followed the optional course wrote a letter of recommendation. Of course, at that time, you would just take holiday leave for such a course, and you paid for it yourself. Meanwhile, I had married a medical student - today she is sitting here on the first row - and I asked the organisers of the course if my wife would be permitted to join me, provided she sat at the back of the hall. The organisers were an American couple – they allowed it.[v] This is how we followed our first course in epidemiology together. When it was over, I had made my decision. Becoming an epidemiologist was the greatest thing in the world.

But how? I found more epidemiology courses, including one that was organised by the Dutch Heart Foundation, taught by a leading academic from Boston, from the Harvard School of Public Health, Olli Miettinen. He presented new viewpoints on old epidemiological notions. At the end of this course, he suggested I come to Boston for further training.

In Boston, I got to know the content of epidemiology. This was one big party. But, I also came across ideas *on* epidemiology. There was talk about a new movement, which was only a few years old, to bring epidemiology back to the clinic. The story that went with it was that a rift had originated between 'Schools of Public Health' and 'Schools of Medicine'. Schools of Public Health had been founded in the 19th century and the beginning of the 20st century. All the numerical thinking about disease and about causes and evolution of frequencies of disease had ended up in the Schools of Public Health. It had disappeared

from the Schools of Medicine. Only basic scientific research and pathophysiological research were still practised there. This had to change – it was said – because medicine was becoming increasingly powerful, expensive and complex. So there had to be an overview in size and number of what happens to patients, as a guideline for what would have to happen in terms of medical intervention. It was deemed necessary to found departments of clinical epidemiology.[vi] The Rockefeller Foundation and the Robert Wood Johnson Foundation supported this. The atmosphere was rife with slogans such as 'Clinical Epidemiology, a basic science for clinical medicine' and 'Clinical Epidemiology, the architecture of clinical medical research'.[vii]

This immediately tied in with my way of thinking: this was it, this was what I wanted to devote myself to.

I enthusiastically informed the faculty in Leuven in 1979; I wanted to found a centre for statistics, epidemiology and informatics – combined – in order to assist clinicians in conducting clinical research. There was no interest. From Boston, Miettinen suggested enquiring in the Netherlands, with the Netherlands Heart Foundation – for there was interest there.

In the Netherlands, medical charity funds were among the first institutions to actively start supporting epidemiology in its clinical application. The then medical director of the Netherlands Heart Foundation, Bart Dekker, had established contacts at Harvard. And so it happened that someone from Harvard *[Miettinen]* came to proclaim the gospel in the Netherlands, in annual courses – of which I had followed the first. Epidemiology at Harvard became the place of pilgrimage. Various epidemiologists and health professionals who were trained in epidemiology from the Netherlands passed through there in the 1980s and 1990s.

I immediately secured a job with the Netherlands Heart Foundation. My main task was to help grant applicants to set up their research better. One of them was Hans Valkenburg, head of epidemiology in Rotterdam – originally an internist and microbiologist, trained in population research in the United States. Valkenburg provided me with a great deal of critical feedback – and I thought him all the more sympathetic for it. After just over a year I switched to Rotterdam.

Valkenburg had been brought to Rotterdam by the founding dean of the Rotterdam faculty, Andries Querido from Leiden. Querido[viii] had become interested in 'community medicine' in the 1960s: you had to understand diseases in the community in which they originate. Upon the foundation of the new Rotterdam faculty, he made sure that a department of epidemiology was included. Later, Querido pleaded for a new stimulus for clinical research - also on the basis of epidemiology (5).

The Rotterdam years turned into five golden years – in continuous consultation with Bert Hofman and Koos Lubsen, we formed a headstrong little group that was going to change the world.

Ideas from the other side of the ocean had an increasingly strong impact. In Amsterdam, Harry Buller was also one of the early converts. He had been trained at MacMaster, Canada, at the brand new department of clinical epidemiology. We charged around the Netherlands with the group of enthusiasts. We organised evenings in university hospitals, with exercises on reading clinical research - not associated with any training structure, without any funding, just in everybody's spare time – because it was so much fun and so new. In 1984, a brainstorming session was organised about the future of clinical epidemiology in the Netherlands, with a representative of the Rockefeller Foundation (6).

This is how all these movements came together. Suddenly, there was interest everywhere. The scarce epidemiologists were offered chairs all over the place. In Leiden, Nol Cats was enthusiastic about the new ideas. Together with Valkenburg, Cats conducted research into the epidemiology of rheumatism. Under their supervision, I gained my doctorate in 1983 with research into the use of oral contraceptives and a possible protection from rheumatoid arthritis (7, 8). Cats paved the way for me in Leiden.[ix] Three years after my doctorate, in September 1986, at the age of 36, I found myself in the brand new building of the current Leiden University Medical Center, all on my own in three rooms, next to the hospital beds storage, sitting on cardboard boxes. This unusual place was the result of the fact that I insisted on being in the hospital, and not somewhere on the other side of the ditch.[x] It had to be easy for clinicians to walk in.

I immediately established contacts with clinical departments in order to find topics that enabled me to demonstrate what the clinical application of epidemiology was good for. Three months after my arrival in Leiden, I met Ernest Briët on the stairs. He told me they had reached a dead end in the research into rare biochemical abnormalities that might be the cause of deep vein thrombosis. Patients who developed thrombosis at an early age, especially when this happened several times in one family, were sent to Leiden from all over the Netherlands for research into biochemical abnormalities. Yet internationally, it was strongly doubted if all of this was that important to the average patient. My immediate response, based on my methodological training, was to argue that they had to stop collecting rare stamps. Rather than conducting research with patients referred from far and wide, I proposed to set up a case-control study with successive patients as they presented in daily practice, as well as with an accompanying control group from the population. In itself, this was a major innovation within coagulation research. The research was set up in intensive cooperation with clinicians and basic scientists. The rest is history: the discovery of factor V Leiden (9), the interaction of factor V Leiden with 'the pill' (10), the whole pill affair with an increased risk of vein thrombosis due to new contraceptives (11), the research into the effect of coagulation mutations in the past, until well in the 19th century (12, 13).

In addition to coagulation, I have concerned myself with a great many other subjects: psychiatry, nephrology, general internal medicine, endocrinology, skin diseases, geriatric medicine and rheumatology. There have been breakthroughs in these areas too – always in collaboration. Such as the research into the presence of antibodies in patients with rheumatoid arthritis, who had been blood donors long before their disease, and in whom the antibodies were detected in their stored blood, a very long time before they developed the disease (14). Diversity is often the fate of the epidemiologist. My hero Greenwood once wrote that he himself had not discovered that much new, but had helped others to discover new things (15). I, too, often look back with a great deal of joy on things that I indirectly made happen, such as the research into the inverse relationship between longevity and fertility – based on historical data from the British nobility (16).

One aspect became gradually clear: the clinical application of epidemiology as it developed in Leiden differed from that of other types of epidemiology. When Evidence-Based Medicine and the Cochrane Collaboration originated at the beginning of the 1990s, the application mainly lay in research into diagnosis, prognosis and therapy. Not in aetiology, the causes - nor in pathogenesis, how these causes interact with the organism. The focus of Leiden epidemiology was precisely these – in particular about disease processes that were of interest to third line physicians. Following a working visit, an American clinical epidemiologist concluded that we practised pathophysiological epidemiology.[xi]

We founded a school, and that was precisely the intention. With Frits Rosendaal, we established the Clinical Epidemiology course on Schiermonnikoog Island, and with Edo Meinders, we set up the epidemiology internship for residents in internal medicine. It was one of the most coveted internships. I have seen many of the residents who followed this internship end up as training supervisors, department heads and chairpersons of national committees. This is not an experimental observation, of course, the residents who chose this internship probably already had it in them, but it is a joyous observation all the same.

In this first part, I have tried to make clear how my career was propelled, mixed up and sometimes severely challenged from all sides in an international movement. This description does not answer the question of *why* numerical thinking broke through in the clinic, and why it did not happen before, in particular at the beginning of the 19th century around the time of Médecine d'Observation (17-19). I would like to further reflect on this with medical historians.

In these developments, I came across a number of problems about which I entered into extensive debates: the battle against *cookbook epidemiology* and the battle for the *soul of science.*

Cookbook epidemiology

Evidence-based Medicine originated from clinical epidemiology around 1990. It was to be a radical break with the past: no longer was medicine to be based on mere expert opinion and on half-baked ideas from basic sciences, but on an objective numerical foundation. The most objective research, it was thought, was the randomised controlled double blind trial. This led to the idea of a hierarchy of evidence, where the randomised trial right at the top was considered to be the pinnacle, and all non-experimental forms of research, such as observational research in its various forms further down, were considered to be increasingly suspect.[xii]

This hierarchy was a thorn in my side from the word go. Medical practice is not based only on results of experiments on diagnostics or therapy. There are a great many important insights that you cannot test with a randomised experiment: think of the transmission of infectious diseases, think of HIV; needless to say, no experiments have been carried out on its transmission with people – and yet we believe observations from which we deduce how this virus is transmitted – global campaigns are based on this knowledge.

My argument was and is that you need all forms of research – *which* type of research depends on the research question. Depending on the research question, all types of research are of equal value (20, 21).

But, ladies and gentlemen, this debate about the hierarchy of evidence is now the debate of the previous century. It has been replaced with a new debate, a new form of simplification that goes much further. This new movement is about 'causal inference' (22). One basic idea of this movement is that you can only make causal statements about actions. Causing something presupposes that you do something, perform an action. This is always the case in a randomised trial; after the randomisation, you set up an action in one group, and a different action in the other group. No problem here. The new movement argues that observational research may be just as good as a randomised trial. This seems an improvement compared to Evidence-Based Medicine. Yet it furthermore argues that observational research can only demonstrate causes when actions are involved. What is wrong with this? Well, one consequence is that what is not an action, but a 'state', can no longer be researched as a cause (23, 24). For example, you can no longer compare the survival rate of a group of fatter people to that of a group of thinner people, and decide that being fat causes a shortened life expectancy, because being fat or thin are not actions. The same would then apply to the state of high blood pressure, or high cholesterol, or carrying a gene – you cannot say anything causal about these. States are not actions and fall outside this so-called 'interventionist view' of causes. There are more views regarding causality, however: there are 'etiological or historical causes' as well (25). These are causes that provide an insight into how something comes about, and this may involve states as well – states in which you probably cannot yet currently intervene – think of carrying a pathogenic gene – but where you might be able to intervene somewhere in the causal path between

gene and disease at some point in the future. The understanding of what a cause is, is far too limited in this new thinking. It is actually not very smart to rely on one type of causality for epidemiology. You need various forms of causality.[xiii]

Why am I fussing so much about these very abstract reasonings on causality? Because in this new thinking, it suddenly looks as though a great deal of research into how diseases originate is no longer legitimate and is now second rate. This grossly disregards the actual power of epidemiology.

The essential problem of cookbook thinking is that solutions are only sought within rules and procedures of one type of knowledge: the methodology of numerical research.[xiv] When you have ticked off all the rules, you may decide that something is a cause. This situation is very similar to that of Baron von Munchausen, who wants to pull himself out of the swamp by pulling on his own hair. This does not work, ladies and gentlemen. In order to get out of the swamp, you need a lever, a lever from outside. This is exactly the same in science. In *all* sciences. The basic scientist who looks for an explanation for the carcinogenic effect of tobacco smoke, does so courtesy of another science: epidemiology. If she did not have epidemiological knowledge about smoking and lung cancer, it would be foolish to search for a carcinogenic mechanism in tobacco smoke (26). Thanks to epidemiology, she can even afford to throw all negative findings away, until she discovers something that seems carcinogenic, for there must be a mechanism. Epidemiology as a science deals with numerical differences between groups of people. Epidemiology also needs levers: numbers may only be collected and interpreted in the light of a story about how things come about. Deciding that something is a cause requires a judgement in which you bring together results from various types of research, and various types of science. This becomes a weighting of methodology, of the exclusion of other explanations, of basic-science and pathophysiological mechanisms. There are no rules for this weighting. The judgement that something is a cause always remains a risk; it is a type of ‘best guess’. This feels uncomfortable. People try to escape this situation with box-checking strategies. But it is what it is: judgement originates through integration and weighting of relevant research and theory. And this is a prelude to the second debate.

The battle for the soul of science.

Science is fragile. Science is fragile for it is based on agreements between people about how you organise the acquisition of knowledge.

The 19th century American philosopher Charles Peirce described that there are various sources of knowledge: tradition, authority, dogma and science. He argued that science is the best source because it means that you jointly search for the right answer, in mutual debate, based on research.[xv] Science only exists when research is published publicly and may be discussed publicly. No research is perfect, and it is possible to criticise any research, but this does not matter – this is the very thing you may argue about. It is precisely because of the imperfection of the previous research that you set up new research. Consensus originates from the discussion; it is never final, is subject to continuous change and is never complete.[xvi] This view of science is a great way of putting things into perspective. Science may be on the wrong track for a long time. Yet we argue that science is 'self-cleansing' in the long run – admittedly, sometimes in the *very* long run. 'Self-cleansing' is *not* to say that we will eventually know how reality works, but it means that ideas that are in conflict with reality will perish in the short or long run. But the rules of the mutual public debate will then have to be observed. A great deal can go wrong here.

Many problems arise under the influence of external factors, where *profit* plays an important role.

This is most clearly visible in research set up by the pharmaceutical industry. Research by one manufacturer nearly always ends up in favour of their own product and to the detriment of the competitor.[xvii] Please note that all research – and this includes basic scientific research – often moves towards what the researcher believes. The reason is that research is 'theory-loaded'; in setting up a study the ideas of a researcher play an important role. This leads to a great many minor and major decisions about the type of patients, the type of outcomes, the way in which you set up the comparison, the duration of the research, etc.; and all these decisions influence the outcome and interpretation of the research (27, 28).

The solution is not just public debate about points of criticism. Public debate is best served by new research that is set up differently by critics of the old research. This is no longer possible for research into products of the pharmaceutical industry, for there is no funding for this – the industry itself exclusively decides what research is carried out with its products. It is not unthinkable that successive new products push out the older ones and are slightly less good in some respects – but that we will never find out. To find out, you need differently set-up research. Please note that this is not to say that the research of the critic always survives and that the research of the industry is always wrong. But in order to know which research survives, both researches have to be present. As research into new

medicines is set up and carried out virtually exclusively by the pharmaceutical industry, a full and worthy debate is no longer possible. Then science ceases to exist (29-31). [xviii]

It is not just about money. Another threat is 'excessive autonomy' thinking. This brought me into debate with health lawyers, ethicists and privacy lawyers. My starting point was that for centuries, physicians have been learning from what they experience with patients; they learn lessons from what occurs to them in practice. This is one of the sources of progress in medicine. To me, the logical result was that this enables and even forces you to use the stored data and material of patients who have already been treated, in order to learn from the experience with previous patients. This idea is at odds with the norm of absolute autonomy where patients need to be asked if this is permitted (32, 33). I went against this based on the principle of solidarity, also with the idea that no personal damage whatsoever is caused to patients by the use of past data, and that patients themselves are enthusiastic about the idea that their data will be used for new insights that may help others. It has repeatedly been demonstrated that research with existing data where individual permission is asked, leads to false results because it is precisely the people who are faced with an unpleasant outcome who respond dismissively when you ask permissions.[xix] In the past decade, this battle has moved to the European level – and has been fought by others in recent years, including Jan Willem Coebergh and the lawyer Evert-Ben van Veen.

The type of open science, as described by Peirce is also under other pressures: by the exceedingly competitive selection mechanisms that are forced on young scientists during grant application processes, because of a dearth of financing - and because more and more often the results of science are not accepted for political, ideological or economic reasons. The central question remains as to how self-evident it is that the ideal of an open science will just continue to exist. It is an agreement about the organisation of knowledge acquisition. This agreement originated in a certain era. It is a cultural expression that requires an effort in order to keep it up, and of which the results are not always easily accepted. Cultural expressions may disappear. It takes vigilance to ensure that they continue to exist (34).

In the third part of this lecture, I would like to say something about the future. As emeritus, you should be very brief about this

In his daydream, Greenwood stated that the clinical application of statistics would take the shape of a diploma, awarded by the Royal Colleges who register medical specialists in England. What is the current situation with the combination of medicine and epidemiology?

Two years ago, the department head of the department of Epidemiology of the Mailman School of Public Health in New York entrusted to me: “We are the last of a dying species”. By ‘dying species’ he meant: the physician-epidemiologist. In his school of public health, there are no longer any physicians who follow the epidemiology masters course, let alone the PhD. In his view, clinical departments need to employ PhD epidemiologists for the supervision of clinical research. Several months ago, by contrast, I was told at the Department of Epidemiology[1] of the University of California in San Francisco that they have set up a separate masters course in epidemiology for clinicians – seemingly in an attempt to turn the tide. And this is also the case at Hopkins and at Harvard.[xx]

It remains my opinion that we need people with both types of knowledge in one head. The same brain understands both worlds: the clinic and methodology. This leads to creativity in the set-up of research and in the development of a new methodology that is required to solve a problem. People with both types of knowledge are able to talk to pure clinicians as well as to pure methodologists, and both conversations will run more smoothly and have a deeper quality – which will lead to better research. Clinicians with methodological knowledge are almost the only ones still capable of reading clinical research in the journals. To the pure clinician, the research from her clinical journals is no longer really understandable, because the set-up and the analysis have become too complicated. People with both types of knowledge are also the ones that can make or break guidelines, for they are able to adopt a critical attitude towards the research behind the guidelines. If we want to preserve and reinforce a proper formation of judgement about the theory and practice of medicine, and for ‘big data’ and for ‘personalised medicine’ as well, then we will need people with both types of knowledge in one head, besides PhD epidemiologists, basic scientists and pure clinicians.

Meanwhile, the large, enthusiastic tidal wave of clinical epidemiology, on which I have surfed over the past 30 years with many others, has stopped raging, and is now quietly lapping on the beach. The future will show what solutions will be selected, for training, for career opportunities as well as for the positioning of clinicians with methodological knowledge.

---

[1] Erratum, original text wrongly stated Department of Public Health; courses for clinicians already exist for a long time at UCSF.

This brings me to my word of thanks.

It is with gratitude that I remember everybody I have been fortunate to know and with whom I have been permitted to co-operate. I have already mentioned many colleagues and friends who shaped me, who brought me here, and with whom I have collaborated. I have worked with too many clinicians to mention them all individually, I am grateful that clinical epidemiology enjoyed such a great reception in the Leiden clinic. I would like to thank Nol Cats and Ernest Briët in particular.

My PhD students and former staff members, from Leiden but from elsewhere as well, in particular from Wageningen, where I taught epidemiology with great pleasure 30 years ago: I thank you for all the cooperation and for the fruitful exchange of ideas. I am proud that 11 of you now hold full professorial chairs yourselves, and that several more will shortly be appointed. And I am no less proud of the fact that nearly half of them are women.

Frits Rosendaal. We have worked together for nearly 27 years. We understand each other with one glance and think the same without needing to consult one another. We performed many missions together, but above all, I like to thank you for all the pleasure.

Frans Helmerhorst, the third of the three musketeers from the illustrious pill affair. Your outspoken opinions and deep friendship have often helped me.

Rudi Westendorp, I thank you for all the sharp exchanges of views, over many years, and for looking over the first version of this lecture.

An unequivocal word of thanks to the Royal Netherlands Academy of Arts and Sciences [Dutch abbreviation: KNAW] who appointed me academy professor in 2006 – thanks to the support of the Leiden University Medical Center and Leiden University. This professorship entailed the obligation to devote oneself entirely to science in complete freedom, to take risks and to pass on knowledge. Sometimes I felt like a latter-day 19th century professor: one who writes in the morning, reads about the subject in the afternoon and continues in the evening with more general subjects, only visiting foreign colleagues to exchange ideas.

I wish to thank many international intellectual sparring partners, from Oxford, Bern, Aarhus, Boston, New York and London, for vigorous and challenging intellectual exchanges. In other academic institutes there is no longer obligatory retirement, so I want to thank in particular Henrik Toft Sørensen for appointing me to Professor of Clinical Epidemiology at Aarhus University, and also Neil Pearce, who is present here today, for constantly plotting to make my stay in London possible.

In 2014, I was presented with the 'Vitaal Bokaal' (a trophy) by the women's network of the LUMC (Vitaal) which is only presented occasionally, and exclusively to men who have

committed themselves to the positioning of women in scientific organisations. I consider this a very great honour; thank you very much for this.

What you achieve is only very partially the result of your own merits – you particularly have to be lucky in terms of health, environment, training and teachers – and these elements have been favourable to me. During his honeymoon, my father[xxi] bought a picture with a poem by Kipling – about organising your life. I quote: *"If you can fill the unforgiving minute with sixty seconds' worth of distance run …"*.[xxii] I still have that picture. The bar was set high – from a medical perspective, a scientific perspective as well as in a selfless commitment to society.

My mother started a very promising scientific career. She abandoned it after my birth – as was usual at the time. Later on, when we did not understand something about genetics at secondary school or at university, she would fetch the large displays of her presentations about her research from the attic to explain it to us. She would drill us with facts and figures at all stages of education and continues to educate us all the time in all respects. She will watch the video recording of this lecture at a later time.

My wife, Christina, and our three daughters, Karianne, Myriam and Annelinde, together with your partners, Daan and Matthias, and children. A great deal has changed, you are now able to continue your own careers, even after building a family, and you are doing this with verve, and with the trial and error that is part and parcel of it. You are doing things I have never done, perhaps because I would not have dared to do them. We share many thoughts and, contrary to previous generations, feelings as well; I learn a lot from you, and indeed increasingly often I need your advice. I am therefore very, very proud of you.

REFERENCES AND NOTES

References

1. Vandenbroucke JP. Klinische Epidemiologie en de geest der Hygiënisten. Utrecht: Wetenschappelijke Uitgeverij Bunge; 1987.

2. Greenwood M. Louis and the numerical Method. IN: The medical dictator and other biographical studies. London: Williams and Norgate; 1936, p. 123-142. Reprinted: The Keynes Press, BMA 1986.

3. Chalmers I, Dickersin K, Chalmers TC. Getting to grips with Archie Cochrane's agenda. BMJ. 1992;305(6857):786-788.

4. Evidence-Based Medicine Working G. Evidence-based medicine. A new approach to teaching the practice of medicine. JAMA. 1992;268(17):2420-2425.

5. Querido A. De 'discipline Geneeskunde' en de nieuwe prioriteiten, weergegeven in het Stimuleringsprogramma Gezondheidsonderzoek 1985. Ned Tijdschr Geneeskd. 1987;131(7):278-284.

6. Danner SA, Müller AS, Vandenbroucke JP. Klinische Epidemiologie, verslag van een workshop. Ned Tijdschr Geneeskd. 1984;128:2280-2282.

7. Vandenbroucke JP. Oral Contraceptives and Rheumatoid Arthritis: further Epidemiologic Evidence for a Protective Effect. Rotterdam: PhD Thesis, Erasmus University Rotterdam; 1983.

8. Vandenbroucke JP, Valkenburg HA, Boersma JW, Cats A, Festen JJ, Huber-Bruning O, et al. Oral contraceptives and rheumatoid arthritis: further evidence for a preventive effect. Lancet. 1982;2(8303):839-842.

9. Bertina RM, Koeleman BP, Koster T, Rosendaal FR, Dirven RJ, de Ronde H, et al. Mutation in blood coagulation factor V associated with resistance to activated protein C. Nature. 1994;369(6475):64-67.

10. Vandenbroucke JP, Koster T, Briet E, Reitsma PH, Bertina RM, Rosendaal FR. Increased risk of venous thrombosis in oral-contraceptive users who are carriers of factor V Leiden mutation. Lancet. 1994;344(8935):1453-1457.

11. Vandenbroucke JP, Rosing J, Bloemenkamp KW, Middeldorp S, Helmerhorst FM, Bouma BN, et al. Oral contraceptives and the risk of venous thrombosis. N Engl J Med. 2001;344(20):1527-1535.

12. Rosendaal FR, Heijboer H, Briet E, Buller HR, Brandjes DP, de Bruin K, et al. Mortality in hereditary antithrombin-III deficiency--1830 to 1989. Lancet. 1991;337(8736):260-262.

13. Allaart CF, Rosendaal FR, Noteboom WM, Vandenbroucke JP, Briet E. Survival in families with hereditary protein C deficiency, 1820 to 1993. BMJ. 1995;311(7010):910-913.

14. Nielen MM, van Schaardenburg D, Reesink HW, van de Stadt RJ, van der Horst-Bruinsma IE, de Koning MH, et al. Specific autoantibodies precede the symptoms of rheumatoid arthritis: a study of serial measurements in blood donors. Arthritis Rheum. 2004;50(2):380-386.

15. Pemberton J. Will Pickles of Wensleydale. The Life of a Country Doctor. Newton Abbot: Country Book Club; 1972, p. 121-122. Reprinted: The Keynes Press, BMA 1983 & RCGP 1984.

16. Westendorp RG, Kirkwood TB. Human longevity at the cost of reproductive success. Nature. 1998;396(6713):743-746.

17. Vandenbroucke JP. Evidence-based medicine and "medecine d'observation". J Clin Epidemiol. 1996;49(12):1335-1338.

18. Vandenbroucke JP. Clinical investigation in the 20th century: the ascendancy of numerical reasoning. Lancet. 1998;352 Suppl 2:SII12-16.

19. Vandenbroucke JP. De opkomst van medische statistiek en epidemiologie in het klinisch wetenschappelijk onderzoek van de afgelopen eeuw. Ned Tijdschr Geneeskd. 1999;143(52):2625-2628.

20. Vandenbroucke JP. When are observational studies as credible as randomised trials? Lancet. 2004;363(9422):1728-1731.

21. Vandenbroucke JP. Observational research, randomised trials, and two views of medical science. PLoS Med. 2008;5(3):e67.

22. Hernán MA, Robins JM. Causal Inference. July 2015:[Available from: http://cdn1.sph.harvard.edu/wp-content/uploads/sites/1268/2015/07/hernanrobins_v1.10.29.pdf.

23. Hernan MA, Taubman SL. Does obesity shorten life? The importance of well-defined interventions to answer causal questions. Int J Obes (Lond). 2008;32 Suppl 3:S8-14.

24. VanderWeele TJ, Hernán MA. Causal effects and natural laws: Towards a conceptualization of causal counterfactuals for nonmanipulable exposures, with application to the effects of race and sex. IN Causality: Statistical Perspective and Applications. Ed: Berzuini C. et al. Hoboken, NJ: John Wiley & Sons, Ltd.; 2012, p. 101-113.

25. Glymour C, Glymour MR. Commentary: race and sex are causes. Epidemiology. 2014;25(4):488-490.

26. Vandenbroucke JP. 175th anniversary lecture. Medical journals and the shaping of medical knowledge. Lancet. 1998;352(9145):2001-2006.

27. Heres S, Davis J, Maino K, Jetzinger E, Kissling W, Leucht S. Why olanzapine beats risperidone, risperidone beats quetiapine, and quetiapine beats olanzapine: an exploratory analysis of head-to-head comparison studies of second-generation antipsychotics. Am J Psychiatry. 2006;163(2):185-194.

28. Smith R. Medical journals are an extension of the marketing arm of pharmaceutical companies. PLoS Med. 2005;2(5):e138.

29. Vandenbroucke JP. Without new rules for industry-sponsored research, science will cease to exist. Rapid response on bmj.com. http://bmjbmjjournalscom/cgi/eletters/331/7529/1350. 2005.

30. Chalmers I. From optimism to disillusion about commitment to transparency in the medico-industrial complex. J R Soc Med. 2006;99(7):337-341.

31. Ioannidis JP. Stealth research: is biomedical innovation happening outside the peer-reviewed literature? JAMA. 2015;313(7):663-664.

32. Van Veen EB, et al. Codering kan beroepsgeheim bij medisch onderzoek waarborgen. NRC-Handelsblad. 8 januari 1994.

33. Appels CW. Vertekening van de resultaten door methode van 'informed consent' bij medisch-wetenschappelijk onderzoek. Ned Tijdschr Geneeskd. 2007;151(12):681-682.

34. McCauley RN. Science's Continued Existence is Fragile. Closing remarks. IN: Why Religion is Natural and Science is Not. Oxford: Oxford University Press; 2013 (Paperback) p. 279-286.

Notes

[i] Major Greenwood (1880-1949). Recent biography: Farewell V, Johnson T. Major Greenwood's early career and the first departments of medical statistics. *Statistics in Medicine* 2014;33:2161-2177.

[ii] Called after Archie Cochrane (1909-1988), writer of the influential book: "Effectiveness and Efficiency: Random Reflections on Health Services", Nuffield Provincial Hospitals Trust, 1972. Biography of Archie Cochrane by Iain Chalmers: *J R Soc Med*. 2008;101: 41–44.

[iii] Prof Dr. JV Joossens (1915-2011), Professor of Cardiology and Head of Department, Academisch Ziekenhuis St Rafaël, Leuven. Obituary: http://www.epi.umn.edu/cvdepi/eulogy-obit/joseph-victor-joossens-1915-2011

[iv] Skjoldenæsholm**,** Copenhagen, 'Ten Day International Teaching Seminar on Cardiovascular Epidemiology', Council on Epidemiology and Prevention of The International Society of Cardiology, and Danish Heart Foundation. Teachers were, amongst others: Rose and Jeremiah Stamler, Geoffrey Rose, Dick Remington, Henry Blackburn.

[v] Jeremiah (1919-) and Rose (1922-1998) Stamler. Biography: https://en.wikipedia.org/wiki/Jeremiah_Stamler

[vi] The origins of this vision about clinical epidemiology in the USA are described by Kerr White: "Healing the Schism: Epidemiology, Medicine, and the Public's Health". Springer, 1991.

[vii] These 'slogans' found their way in the titles of two leading textbooks:
- Feinstein AR. "Clinical Epidemiology: The Architecture of Clinical Research. Saunders, 1985.
- DL. Sackett, R. B Haynes, H Guyatt, P Tugwell. Clinical Epidemiology a Basic Science for Clinical Medicine." Little Brown and company, Boston, 1985 (2nd edition: 1991). [Biography David Sackett (1934-2015): BMJ 2015;350:h2639 doi:10.1136/bmj.h263]

[viii] Andries Querido (1912-2001). The role of Querido in the history of epidemiology at Rotterdam is described in: A Hofman, Veertig jaar epidemiologie aan de Erasmus Universiteit 1969-2009. Erasmus Universiteit. ISBN 978-90-8559-607-3

[ix] To facilitate the founding of a department of clinical epidemiology, the Leiden Medical Faculty decided to disband the department of social medicine after the retirement of its chair. The background to this decision is described in a brief by Mr. C.J. Zwarts, Director of the Faculty, April 1984

[x] A ditch separated clinical and non-clinical institutes at the time in Leiden.

[xi] Alvan Feinstein (1925-2001). The *Journal of Clinical Epidemiology* published an obituary issue in December 2012, which also included papers written by persons who did not agree with his teachings.

[xii] Later, several "Hierarchies of Evidence" have been proposed; in all of them the randomized trial ranks above observational research. Sometimes meta-analyses of randomized trials, or n=1 trials are ranked above randomized trials.

[xiii] The new causal thinking in epidemiology also disregards the fact that deciding about causality almost always requires more than a piece of research that emulates an experiment. A cornerstone of epidemiologic thinking is 'ruling out alternative explanations'. (See: Cornfield J. Principles of Research. *American Journal on Mental Deficiency, 1959;64, 240–252.* Reprinted in *Stat Med 2012,* with several commentaries). For example, against research showing that smoking during pregnancy leads to reduced growth in offspring, critics may argue that this might not be an effect of smoking itself, because smoking mothers could differ in many respects from non-smokers: in socio-economic class, in abiding to all kinds of rules during pregnancy, etc. One argument against such criticism is research about smoking by fathers and its impact on growth of their offspring; the effect of smoking by fathers is almost zero (Howe et al. Maternal smoking during pregnancy and offspring trajectories of height and adiposity: comparing maternal and paternal associations. *International Journal of Epidemiology* 2011;41:722-732). This is a strong argument that the effect is not due to all kinds of other living conditions of the mothers, but that their smoking is truly a cause to this effect. None of the individual pieces of research is sufficient by itself, but their combined interpretation makes other explanations less credible.

[xiv] Cookbook thinking also led to the misconception that research is only good if there is complete preplanning in a completely worked-out protocol that is strictly adhered to during execution. This is an excellent idea in the case of randomized trials which are intended to lead to the licensing of a drug. Then it is a procedural guarantee for the licensing authorities. However, it is nonsensical to apply this principle to research trying to explain how nature works; in such research one tries to find an explanation for a problem and one thinks continuously of new solutions for the problem. In such circumstances, it is unimportant to know with what thoughts the research started and which thoughts developed in the course of doing the research. (Lash TL, Vandenbroucke JP. Should preregistration of epidemiologic study protocols become compulsory? *Epidemiology* 2012;23: 184–188). The only thing that counts is the final public proposal to fellow scientists : an explanation and the associated research data – it enables them to decide whether this constitutes a basis for future research.

[xv] According to Charles Sander Peirce (1839-1914) science is in principle superior for theoretical knowledge; for other types of skills, other types of knowledge may be more valuable. See a potted description of his views under the subheading "*Rival modes of inquiry*" IN: https://en.wikipedia.org/wiki/Charles_Sanders_Peirce .

[xvi] Max Weber (1864-1920) wrote in his *"Wissenschaft als Beruf"* in 1918 (a title diversely translated as "Science as a vocation" and "Science as a profession", and actually having both meanings) that scientists know that their theories and their results will be temporary because they will be amalgamated in new theories and new results, and will thereby disappear. According to Weber that is the essence of science. See: http://www.wisdom.weizmann.ac.il/~oded/X/WeberScienceVocation.pdf (page 8 bottom).

[xvii] This leads to ridiculous situations, such as the finding that in a head-to-head comparison between drug A and drug B, drug A proves to be superior, while in the comparison between B and C, B is superior, but that in the direct comparison between C and A, drug A was the best – the findings are apparently guided by who happened to be the sponsor of the studies. See references 27 and 28.

[xviii] A new problem is the establishment of 'businesses' by academic researchers, to further develop their findings into marketable products. Partly due to the influence of capital raised from the outside, these 'businesses' tend to behave in the same way as a pharmaceutical industries (Vandenbroucke JP. Waarom het einde van de wetenschap nadert. *Opiniepagina NRC-Handelsblad* 21-08-2007).

[xix] International studies that have demonstrated this bias are given in reference 33.

[xx] A sample from different programs:
- University of California at San Francisco, Training in Clinical Research (TICR) Program (http://www.epibiostat.ucsf.edu/courses/post.html);
- Johns Hopkins Bloomberg School of Public Health, Graduate Training Programs in Clinical Investigation (http://www.jhsph.edu/academics/graduate-training-programs-in-clinical-investigation/);
- Harvard TH Chan School of Public Health, Summer Program in Clinical Effectiveness (http://www.hsph.harvard.edu/clinical-effectiveness/)

[xxi] Prof Dr. J. Vandenbroucke (1914-1987): Biography by K. Geboes, 'Vandenbroucke, arts, specialist in de inwendige geneeskunde, geaggregeerde van het Hoger Onderwijs, Diensthoofd-Directeur Inwendige Geneeskunde Universitaire Ziekenhuizen K.U.L. (1952-1987)', in: Nationaal Biografisch Woordenboek, Koninklijke Academie voor Geneeskunde van België 2009;19:961-977.

[xxii]The poem "If" by Rudyard Kipling, 1909
